# Outcomes of antibiotic therapy and transvaginal ultrasound-guided efficacy of transvaginal ultrasound-guided drainage in treating tubo-ovarian abscesses: Three case reports

**DOI:** 10.18502/ijrm.v22i11.17825

**Published:** 2025-01-10

**Authors:** Zahra Heidar, Tayebeh Esfidani, Atefeh Moridi, Mahtab Anvari

**Affiliations:** Clinical Research Developement Center, Mahdiyeh Educational Hospital, Shahid Beheshti University of Medical Sciences, Tehran, Iran.

**Keywords:** Abscess, Ovarian, Drainage, Ultrasonography, Endometrioma.

## Abstract

**Background:**

To introduce minimally invasive methods for the successful treatment of tubo-ovarian abscesses (TOAs), an antibiotic regimen was considered the first line of treatment. However, in some cases, this approach fails, and another intervention (laparotomy or minimally guidance drainage) is required.

**Case Presentations:**

3 women with a history of long-time infertility, all of them were candidates for in vitro fertilization referred to the obstetrics and gynecology department with similar manifestations. For these 3 cases (30–40 yr) the first approach was a broad-spectrum antibiotic therapy. In 2 cases the last step in treatment was transvaginal ultrasound guidance drainage, and in one case laparotomy was done after antibiotic regimen failure; however, in all of 3 cases the best results were seen in transvaginal ultrasound guidance drainage.

**Conclusion:**

Patients who have ovarian endometrioma and undergo an assisted reproductive technology cycle, as well as ovum pick up, increase the possibility of TOA occurrence in them. The use of transvaginal ultrasound guidance drainage approach for the treatment of TOA in selective cases, in addition to broad-spectrum antibiotics in patients might reduce their need for invasive treatment with laparotomy.

## 1. Introduction

A tubo-ovarian abscess (TOA) is a complex infectious mass in the adnexa that forms as a consequence of pelvic inflammatory disease (PID). Classically, TOA presents with an adnexal mass, fever, increased white blood cell (WBC) count, and lower abdominal pelvic pain and/or vaginal discharge (1). TOAs are a relatively common complication, involving approximately 10–15% of women with PID (2, 3).

These abscesses are most commonly found in reproductive-age patients, although they can also occur in sexually inactive teenage girls or postmenopausal women. Previously, we considered that only the 2 microbial agents, *Neisseria gonorrhea* and *Chlamydia trachomatis* are the cause of TOAs (4, 5). However, it has now been proven that the polymicrobial factors are responsible for this situation (6). TOA typically occurs as a complication of PID; in addition, it may be caused by a variety of conditions including diverticulitis, or even inflammatory bowel diseases, and from the spread of infection to nearby organs usually the appendix, and less commonly via blood spread from a more distant site of infection, or as an association with pelvic organ cancer (7, 8).

Several cases of TOA following oocyte retrieval for in vitro fertilization have been reported. Inpatients with ovarian endometriomas, TOAs can occur even long after the completion of the assisted reproductive technology (ART) cycle. The first step in managing TOAs is intravenous (IV) prescription of broad-spectrum antibiotics targeting gram-negative aerobes and anaerobes. According to the previous literature, this regimen provides a success rate of 75–85% (9, 10). Thus, in the last decades, minimally invasive drainage techniques have been suggested and performed even as the first line, but not as the only treatment option (7).

Previously published studies have reported a success rate of 78–100% for transvaginal ultrasound-guided drainage of TOAs. Also, another approach is transabdominal (computed tomography) CT-guided or ultrasound-guided drainage of TOAs with a reported success rate of about 95%. Currently, there are no precise guidelines for choosing the best treatment options for TOA patients (11).

In the present case report, we report cases of TOAs in 3 women with a history of infertility, which needed minimally invasive interventions following failure in antibiotic therapy alone.

It is important to mention that all the studied patients were selected among the individuals referred to Mahdieh hospital in Tehran, Iran. Also, the patients were examined by 3 gynecologists. From 2014–2023, about 180 patients with ovarian endometrioma underwent ovarian puncture on ultrasound, of which 60 had bilateral ovarian endometrioma.

## 2. Case Presentations

### Case 1

A 32-yr-old nulliparous woman, body mass index (BMI): 27 (kg/m^2^), with a history of 6 yr of infertility, stage 4 endometriosis, 2 sessions of laparoscopy in 2011 and 2012, as well as right tubectomy due to hydrosalpinx, had undergone in vitro fertilization in 2014; following stimulation of ovulation, 3 follicles were developed. On the ovarian puncture day, 2 oocytes were retrieved, but the patient attended our clinic with chief complaints of nocturnal fever, abdominal pain, intermittent nausea, and anorexia 2 wk after the procedure. She had no symptoms of urinary tract infection or dyspareunia. The last session of sexual intercourse was 15 days before attendance.

On physical examination, no abdominal rigidity or tenderness was found; however, there was a slight abdominal distension. She had an oral temperature of 38.2 C. On ultrasound, both ovaries had increased in diameter with normal vascular flow. Also, a 30 × 40 mm cystic lesion in the right ovary was reported, suggestive of endometrioma. A few free fluids were detected in the pelvic, as well as spleen-renal and pleural spaces. No evidence of abscess was seen. In addition, leukocytosis was observed in laboratory tests (WBC: 24.3 × 1000 cells/µL). The patient underwent antibiotic treatment (piperacillin-tazobactam 3.75 gr IV, 3 times daily [TdS] and vancomycin 1 gr, IV, twice in a day [BID]).

According to persistent abdominal pain and fever on the 7 ${\;}^{\text{th}}$ day of admission, an abdominal CT scan was performed which indicated a 147 × 43 mm abdominal collection beside the right tubo-ovarian region and medial to small intestine. Also, an echocardiography was performed which reported no evidence of endocarditis. The patient underwent laparotomy for resection of endometrioma and drainage of the abscess. In the right adnexal region, a 50 × 70 mm collection was seen around the right ovary. Also, a 20 × 30 mm endometrioma was found in the right adnexal region. The collections were drained, and 2 drains were placed in the region on the right and left sides.

After 72 hr of laparotomy due to persistent fever, a transvaginal ultrasound was performed and 3 collections with a size of 25 × 25 mm, 30 × 15 mm, and 20 × 20 mm were seen; therefore, the collections were drained under transvaginal guidance. On the same day (72 hr after laparotomy), a left drain was removed due to the absence of additional discharge. 48 hr after drainage under transvaginal guidance (5 days after laparotomy), transvaginal ultrasound assessments were performed which showed the absence of further collections, hence the right drain was removed. An antibiotic regimen including Meropenem (1 gr, IV, TdS), Amikacin (300 mg, IV, TdS), Metronidazole (500 mg, IV, TdS), as well as 5000 units of (sub cutaneous) SC Heparin (BD), was started initially for the patient. The patient had an improving trend in clinical manifestations (WBC: 7000 cells/µL) after 25 days of admission and was finally discharged in good and stable condition with the oral antibiotic regimen, including Ciprofloxacin (500 mg, oral administration [PO], BID) and Amoxicillin-Clavolunate (625 mg, PO, TdS). The initial antibiotic regimen (Vancomycin and Piperacillin-tazobactam) in this patient was changed to Meropenem, Vancomycin, and Metronidazole with the opinion of the infection specialist due to the continued fever and sensitivity to Vancomycin (hives and rash). During 1 yr follow-up, no evidence of recurrence of abscess or fever and pelvic infection was observed in the patient.

### Case 2

In December 2019, a 40-yr-old nulligravid woman, BMI: 28.3 (kg/m^2^), with a history of 10-yr infertility and a history of 2 times intra cytoplasmic sperm injection cycles and oocyte retrieval procedures, visited our clinic. She had no other medical history. She had a chief complaint of anorexia and abdominal pain for a week, which has been exacerbated since the last night of the visit. The pain was in a hypogastric area with radiation to the right inguinal region. She had no complaints of nausea, vomiting, diarrhea, or weight loss. She had a history of surgery for an ovarian cyst and left salpingectomy due to salpingitis about 10 yr ago.

On physical examination, she had an oral temperature of 38.3 C, in laboratory tests leukocytosis was found, (WBC: 23 × 1000 cells/µL). No abdominal guarding or rigidity were observed; however, she had a slight tenderness in right lower quadrant of the abdomen and fullness in the posterior choledosac. On bimanual examination, a mass was palpable indicating 16 wk of gestational age. In the second case, although the patient had a history of ovarian puncture in the previous 3 yr, she did not have a clear history of endometriosis, but the patient had a TOA.

The patient underwent an ultrasound evaluation. The uterus had normal size and echogenicity. An 81 × 60 mm multicystic lesion was detected superior to the uterus with extension to the right adnexal region. A tubular region with increased vascularity and a diameter of 26 mm was seen within this legion suggestive of pyosalpinx. An echo-free fluid was seen in the posterior. After this evaluation an antibiotic regimen including clindamycin (900 mg, IV, TdS) and Gentamycin (320 mg, IV, daily) was started for the patient; however, it was discontinued due to diarrhea and Intravenous Ampicillin-Sulbactam (3 gr, IV, 4 times a day, QID), Levofloxacin (500 mg IV, daily), and Vancomycin (1 gr, IV, BD) was started.

Due to persistent fever after 48 hr, the cystic purulent lesion was drained with transvaginal ultrasound guidance, and a drain was placed. After one day the patient had no more fever following drainage. The drain was removed due to lack of additional discharges, and on day 4 of its placement Levofloxacin was changed to oral form (500 mg, daily). Also, Vancomycin and Ampicillin-Sulbactam were discontinued, and Metronidazole (500 mg, PO, BD) was started. The patient was discharged in good condition (WBC: 7.2 × 1000 cell/ml, T: 37.3) with the following oral antibiotic regimen: Levofloxacin 500 mg daily, Cloxacillin 500 mg QID, and Metronidazole 500 mg BD. No adverse events occurred during the 1 yr follow-up of the patient.

### Case 3

A 34-yr-old woman, gravid 1 ectopic pregnancy 1, BMI: 26 (kg/m^2^) with a history of hypertension and right tubal salpingectomy due to ectopic pregnancy 15 yr ago, infertility, and as a candidate of in vitro fertilization (following stimulation of ovulation 4 follicles were developed) referred to the clinic. On the ovarian puncture day, 3 oocytes were retrieved.

2 ovarian endometrioma with a size of 40 × 35 mm and 30 × 35 mm in the right ovary and 25 × 30 mm in the left ovary were seen, located near follicles that were not ruptured during the ovarian puncture procedure. However, antibiotic regimen was started (Metronidazole, 500 mg PO, TdS, and Cefixime 400 mg PO, daily), and after 4 days, 2 fetuses were transferred. The patient had not taken her medicines, she was admitted with the symptoms of abdominal pain, fever (oral temperature of 38.1 C), nausea, weakness, and lethargy on September 2023 (2 wk after oocyte retrieval). On physical examination no abdominal rigidity or tenderness was found, leukocytosis was obvious in the laboratory test (WBC: 22 × 1000 cell/µL) and the β-human chorionic gonadotropin test was negative.

In transvaginal ultrasonography an hypohetero-echo tubular structure lesion with a size of 121 × 132 × 120 mm and a volume of about 1000 cc inside the pelvic cavity, in the vicinity of the uterus in favor of hematosalpinx or pyosalpinx was evident. Antibiotic therapy with Metronidazole (500 mg, IV, TTS), Ceftriaxone (1 gr, IV, BD), and Doxycycline (100 mg, PO, BD) was started. After 48 hr of antibiotic therapy, the fever continued, the transvaginal ultrasonography was performed again, and several abscesses with a size of 132 × 80 × 48 mm space, 136 × 103 × 97 mm and 50 × 45 × 30 mm in the right adnexa, and 63 × 92 × 97 mm in left adnexa with extension to the posterior cul de sac were seen.

Ceftriaxone was changed to Meropenem (1 gr, IV, TdS) and Vancomycin (1 gr, IV, BD), Metronidazole (500 mg, IV, TdS), and Doxycycline (100 mg, PO, BD) were continued. The abscess was drained by transvaginal ultrasound guidance and a drain was placed. No evidence of intestinal perforation in the Abdominopelvic CT scan was observed. Acinetobacter was isolated from secretion culture; therefore, Amikacin (300 mg, IV, TdS) was added to the antibiotics, and the second and the third transvaginal ultrasound guidance drainage in the 5 ${\;}^{\text{th}}$ and 10 ${\;}^{\text{th}}$ days of the first drainage were performed, respectively. Subsequent drainage cultures were negative. The patient had an improving trend in clinical manifestations (T: 37.1, WBC: 5.4 × 1000 cells/µL).

Due to the absence of fever within 72 hr and good general condition, she was discharged with an oral antibiotics regimen, including Ciprofloxacin, 500 mg, PO, BID, and Clindamycin 300 mg PO, TDS. No adverse events occurred in the 6-month follow up.

### Ethical Considerations

Written informed consent was obtained from the patients publishing this case series report. A copy of the written consent is available for review with the Editor-in-Chief of this journal.

## 3. Discussion

TOA is potentially a life-threatening condition due to the risk of TOA rupture and sepsis that can quickly lead to overwhelming sepsis and death (9). According to past studies, factors such as ART, older age, intrauterine contraceptive devices, postmenopausal STI, and endometriosis (with ovarian endometrioma) increase the risk of TOA, especially after ovum pick up (OPU) procedure (12, 13).

In the present study, we reported 3 cases of TOAs in patients with a positive history of infertility and OPU procedure, 2 cases had a history of endometrioma, and TOA occurred after 2 wk of OPU.

In these 3 cases, the first approach was broad-spectrum antibiotic therapy; however, all cases needed minimally invasive procedures (ultrasound-guided drainage of abscess). One case underwent laparotomy due to persistent fever and relative failure of antibiotic therapy, but finally, there was an improvement with transvaginal ultrasound guidance drainage. All cases were discharged with an oral antibiotic regimen, shortly after minimally invasive interventions with no more evident long-time complications. In all 3 cases, the best results were seen in transvaginal ultrasound guidance drainage.

Current international guidelines hold the belief that medical treatment including broad-spectrum antibiotic therapies should be taken into consideration for the treatment of TOAs in hemodynamically stable patients as the first line (14). On the other hand, previously published studies have reported a wide range of success rates for antibiotic therapy alone; the highest rate is to be about 85% (6). Nowadays, interventional radiology experts and procedures are widely available nearly all around the world. However, it still remains a controversy, as to which TOA patients will take the most benefits from medical treatment alone and which one will need simultaneous radiological intervention, such as image-guided drainage, in addition to antibiotic therapy.

A clinical study reported that patients with TOAs > 35 mm failed to respond to IV antibiotic treatment and needed a kind of radiologic intervention (6), which is in agreement with our results. Subsequent investigations have noted that the size of the abscess predictive of necessitating drainage is more likely in the range of 70 mm (2).

In our study, the best results were seen in transvaginal ultrasound guidance drainage where all our cases had an abscess size of more than 70 mm.

Another study evaluated 122 patients with TOA of which a majority (65.6%) were treated by medical regimen alone. They reported that larger size of TOA, older age, and higher parity rates were associated with failure of antibiotic therapy; while all of our 3 patients were infertile till the time of admission (15). This disagreement may be due to the difference in study design and the limited sample size of our study.

Previous study showed that the use of antibiotic therapy along with image-guided drainage can reduce complications and also reduce the length of hospitalization of patients compared to laparoscopic drainage (16). In another study, showed that the use of surgical methods compared to drug treatments (use of antibiotics) can be associated with faster recovery of patients (17).

In the present study, all the patients with TOA did not respond to the initial broad-spectrum antibiotic therapies despite various treatment regimens, which were prescribed through infectious disease service consultations.

## 4. Conclusion

In general, it can be said that patients who have ovarian endometrioma and undergo ART cycle as well as OPU, increase the possibility of TOA occurrence in them; the use of transvaginal ultrasound guidance drainage approach for treatment of TOA in selective cases, in addition to broad-spectrum antibiotics in patients reduces their need for invasive treatment with laparotomy.

##  Data Availability

Data supporting the findings of this study are available upon reasonable request from the corresponding author.

##  Author Contributions

Z. Heidar: Design and conduct the study. T. Esfidani and A. Moridi: Critically revised the manuscript. M. Anvari: Interpreted the findings and drafted the manuscript. All authors approved the final version of the manuscript.

##  Conflict of Interest 

The authors declare that there is no conflict of interest.
